# Molecular characterization of multidrug-resistant extended-spectrum β-lactamase-producing *Enterobacteriaceae* isolated in Antananarivo, Madagascar

**DOI:** 10.1186/1471-2180-13-85

**Published:** 2013-04-17

**Authors:** Hanitra C Rakotonirina, Benoît Garin, Frédérique Randrianirina, Vincent Richard, Antoine Talarmin, Guillaume Arlet

**Affiliations:** 1 Institut Pasteur de Madagascar, Antananarivo 101, BP 1274, Madagascar; 2 Institut Pasteur de Dakar, 36 Avenue Pasteur, Dakar BP 220, Senegal; 3 Institut Pasteur de Guadeloupe, Morne Jolivière, 97183 Les Abymes, Guadeloupe, BP 484, France; 4 Faculté de Médecine, Laboratoire de Bactériologie, Université Pierre et Marie Curie, Paris 6, 27 rue Chaligny, Paris 75012, France; 5 Assistance Publique Hôpitaux de Paris, Hôpital Tenon, Laboratoire de Bactériologie, 4 rue de la Chine, Paris 75970, France

## Abstract

**Background:**

We investigated the molecular characteristics of multidrug-resistant, extended-spectrum β-lactamase (ESBL)-producing *Enterobacteriaceae* isolated in community settings and in hospitals in Antananarivo, Madagascar.

**Results:**

Forty-nine *E. coli, K. pneumoniae, K. oxytoca* and *E. cloacae* ESBL-producing isolates were studied. In antimicrobial susceptibility analyses, many of the isolates exhibited resistance to aminoglycosides, fluoroquinolones and trimethoprim-sulfamethoxazole. Gene amplification analysis and sequencing revealed that 75.5% (n=37) of the isolates harbored *bla*_CTX-M-15_ and 38.7% (n=19) harbored *bla*_SHV-12_. The non-ESBLs resistance genes detected were *bla*_TEM-1_, *bla*_OXA-1_, *aac(6*^*′*^*)-Ib,aac(6*^*′*^*)-Ib-cr, tetA*, *sul-1*, *sul-2*, *qnrA, qnrB* and *catB-3*. We found *dfrA* and *aadA* gene cassettes in the class 1 integron variable regions of the isolates, and the combination of *dfrA17-aadA5* to be the most prevalent. All *bla*_CTX-M-15_ positive isolates also contained the *ISEcp1* insertion element. Conjugation and transformation experiments indicated that 70.3% of the antibiotic resistance genes resided on plasmids. Through a PCR based replicon typing method, plasmids carrying the *bla*_SHV-12_ or *bla*_CTX-M-15_ genes were assigned to either the IncFII replicon type or, rarely, to the HI2 replicon type. All isolates were subtyped by the rep-PCR and ERIC-PCR methods.

Phylogenetic grouping and virulence genotyping of the *E. coli* isolates revealed that most of them belonged to group A1. One isolate assigned to group B2 harbored *bla*_CTX-M-15_ and five virulence genes (*traT*, *fyuA*, *iutA*, *iha* and *sfa*) and was related to the O25b-ST131 clone.

**Conclusions:**

Our results highlight the dissemination of multidrug resistant *Enterobacteriaceae* isolates in Antananarivo. These findings underline the need for a rational use of antibiotic and for appropriate methods of screening ESBL in routine laboratories in Antananarivo.

## Background

Extended-spectrum β-lactamase (ESBL)-producing bacteria represent a major worldwide threat among drug-resistant bacteria in both hospital and community settings [1]. ESBLs are among the Ambler classes A, confer resistance to β-lactam antibiotics except cephamycins and carbapenems, and are inhibited by clavulanic acid [1]. ESBLs are often located on large plasmids that also harbor resistant genes to other antimicrobial classes with resulting multidrug-resistant isolates [2].

The first ESBLs have evolved by genetic mutation from native β-lactamases TEM and SHV [3][4]. Recently, a novel type of ESBLs, the CTX-M enzymes, emerged worldwide, mostly from *Enterobacteriaceae*[5,6]. CTX-M β-lactamases are not closely related to TEM or SHV ESBLs but share high amino-acid identity with chromosomal β-lactamases from *Kluyvera* spp. [7]. Now, *bla*_*CTX*-M-15_ is recognized as the most widely distributed CTX-M enzyme [8]. It is derived from CTX-M-3 by a substitution of Asp-240-Gly which increases its catalytic efficiency against ceftazidime [9]. *bla*_CTX-M-15_ are encoded on plasmids belonging to the incompatibility group IncF [10]. In the upstream region of CTX-M genes an insertion sequence element, *ISEcp1,* is commonly present and is likely responsible for the transposition process of the genes [11].

*E. coli* is among the most prevalent causes of hospital-acquired and community-acquired bacterial infections and their resistances to antimicrobial agents have become a serious concern for healthcare providers [5]. Phylogenetic analyses have classified *E. coli* into four main phylogenetic groups (A, B1, B2, and D). Commensal isolates belong mainly to A and B1 groups whereas virulent extra-intestinal pathogenic *E. coli* (ExPEC) are essentially from the B2 and D groups [12,13]. ExPEC harbor numerous virulence factors including α-hemolysin, cytotoxic necrotizing factor, adhesins and iron acquisition systems [12]. The spread of *bla*_CTX-M-15_ has been mainly associated with the dissemination of a particular clone of *E. coli* ST131 belonging to phylogenetic group B2 [14,15]. Recently, an *E. coli* clone O25 ST131, producing CTX-M-15, with high virulence potential and belonging to the B2 group, has been reported and represent a major public health problem [14,15].

Many reports have documented the emergence of ESBL-producing *Enterobacteriaceae*[16-18]. In Antananarivo, ESBLs were first detected in 2005 from UTI in 9.7% of isolated *Enterobacteriaceae*[19]. In 2006, outbreaks of CTX-M-15 and SHV-2-producing *K. pneumoniae* isolates have been described in two pediatric units [20]. More recently, 21.3% of clinical isolates from patients in surgery and intensive care units [21] and 21.2% of intestinal carriage isolates from children hospitalized in a pediatric department of a large teaching hospital [22] were ESBL-producers.

For 49 multidrug-resistant *Enterobacteriaceae* isolates from Antananarivo, we characterized: i) the genes encoding the ESBLs; ii) the drug resistance genes associated with the ESBL genes; iii) gene cassettes present in the isolates; and iv) the plasmid incompatibility groups of the isolates. We also determined the phylogenetic groups and virulence factors of the *E. coli* isolates.

## Methods

### Ethical clearance

The study protocols were approved by the National Ethics Committee of Madagascar. Written informed consents were obtained from all patients and at least one parent of each child before enrollment.

### Patients

Between September 2006 and December 2007, a total of 909 non-duplicate bacterial isolates were obtained from 909 patients. 830 patients were recruited from several wards in four hospitals in Antananarivo, Madagascar (two national university teaching hospitals: Joseph Ravoahangy Andrianavalona Hospital and Befelatanana Hospital; a military hospital: Soavinandriana Hospital; and a pediatric hospital: Tsaralalana Hospital) and 79 patients referred to the Pasteur Institute Medical Laboratory in Antananarivo.

### Laboratory methods

Various clinical specimens (including blood-culture, urine, pus, sputum and CSF) were collected and submitted for bacterial analysis at the Pasteur Institute Medical Laboratory in Antananarivo. Presumptive Enterobacteria isolates were identified using standard microbiological methods and the API 20E system (Bio-Mérieux SA, Marcy l’Etoile, France).

### Antimicrobial susceptibility testing and ESBL detection

Antimicrobial susceptibilities were determined by the disk diffusion method on Mueller-Hinton agar (Bio-Rad, Marne la Coquette, France) according to the guidelines of the *Comité de l’antibiogramme de la Société Française de Microbiologie*. The following antibiotics were tested: amoxicillin, amoxicillin-clavulanate, ticarcillin, cephalotin, cefamandole, cefoxitin, cefotaxime, ceftazidime, imipenem, gentamicin, tobramycin, netilmicin, amikacin, nalidixic acid, pefloxacin, ciprofloxacin and trimethoprim-sulfamethoxazole.

Suspected ESBLs were confirmed by the double-disk synergy test. *E. coli* ATCC 25922 and *K. pneumoniae* ATCC 700603 were used as quality control strains.

### Fingerprinting analysis

After DNA extraction by using the Qiagen Mini kit (Qiagen, Courtaboeuf, France), repetitive extragenic palindromic (Rep-PCR) and Enterobacterial repetitive intergenic consensus sequence PCR (ERIC-PCR) were performed with the rep-1R, rep-2 T and ERIC-2 primers, respectively, as previously described [18]. Pattern profiles were considered different when at least one band differed.

### Molecular characterization of resistance genes

DNA was extracted by the boiling method. ESBL-encoding genes were identified using specific primers for the *bla*_TEM_, *bla*_SHV_, *bla*_CTX-M_ and *bla*_OXA_ genes, previously described [23], and followed by DNA sequencing. Other *bla*_CTX-M-15_-associated antibiotic resistance genes (i.e., *aac(6*^*′*^*)-Ib,qnrA, qnrB, qnrS, tetA, sul1* and *sul2*) were screened by PCR [24,25]. All positive isolates for the *aac(6*^*′*^*)-Ib* gene were further analyzed by digesting the purified PCR products with *BtsCI* (New England Biolabs, Beverly, MA) to identify *aac(6*^*′*^*)-Ib-cr,* which lacks the *BtsCI* restriction site present in the wild-type gene [26]. The upstream sequence of the *bla*_CTX-M_ genes was explored by PCR and sequenced to detect *ISEcp1*. The integrase gene (*int*1) was detected by PCR using specific primers [27]. The variable region of each class 1 integron was amplified using specific primers for the 5^′^ conserved segment (5^′^CS) and 3^′^ conserved segment (3^′^CS) [27], and gene cassettes were sequenced. BlastN was used to compare the sequences obtained to those present in the GenBank database (http://blast.ncbi.nlm.nih.gov).

### Resistance transfer assays

Conjugations were carried out in trypticase soy broth (Bio-Rad), with *E. coli* J53-2 (*pro, met,* Rif^r^) as the recipient. Mating broths were incubated at 37°C for 18 hr. Transconjugants were selected on Drigalski agar plates (Bio-Rad) containing rifampicin (250 μg/ml) and cefotaxime (2.5 μg/ml).

Transfer experiments using electroporation were performed for non-conjugative plasmids. Plasmid DNA from donors was extracted with a QIAGEN plasmid midi kit (QIAGEN, Courtaboeuf, France). Purified plasmids were used to transform *E. coli* DH10B (Invitrogen SARL, Cergy-Pontoise, France) by electroporation following the manufacturer’s instructions (Bio-Rad). Transformants were incubated at 37°C for 1.5 hr and then selected on Drigalski agar (Bio-Rad) supplemented with 2.5 μg/ml cefotaxime.

Transconjugants and transformants were tested for ESBL production followed by PCR amplification of the ESBL genes and plasmid replicon typing.

### Plasmid replicon type determination

Plasmid replicons from transconjugants and transformants were determined using the PCR-based replicon typing method described previously by Carattoli *et al*. Eighteen pairs of primers targeting the FIA, FIB, FIC, HI1, HI2, I1, L/M, N, P, W, T, A/C, K, B/O, X, Y, F and FII replicons were used in single or multiplex PCR [28].

### Phylogenetic group and virulence genotyping of *E. coli*

The phylogenetic groups of the *E. coli* isolates were determined by PCR, [13], using a combination of three DNA gene markers (*chuA, yjaA* and *TSPE4-C2*). All isolates belonging to group B2 were analyzed by duplex PCR targeting the *pabB* and *trpA* genes to determine whether the isolate was a member of the O25b-ST131 clonal group or not [29].

The presence of 15 virulence factors found in ExPEC was investigated by PCR with primers reported previously [16]. These factors included *fimH* (type 1 fimbriae)*, sfa/foc* (S and F1C fimbriae)*, papG* alleles (G adhesin classes of P fimbriae), *afa* (fimbrial adhesin)*, hlyA* (alpha-haemolysin A)*, cnf* (cytotoxic necrotizating factor 1)*, fyuA* (genes of yersiniabactin), *iutA* (aerobactin receptor), *kpsMII* (group 2 capsules)*, traT* (genes related to complement resistance), *sat* (secreted autotransporter toxin)*, IroN* (iron related genes) and *Iha* (IrgA homologue adhesin).

## Results

### Description of the bacterial isolates

During the study period, we collected 909 isolates, of which 830 from hospitalized patients and 79 from patients attending the Pasteur Institute medical laboratory. Among these, 262 were identified as *E. coli* (n=75)*, K. pneumoniae* (n=95)*, K. oxytoca* (n=12) or *E. cloaca*e (n=80) and 239 were ESBL-producers of which 49 were selected for in-depth analysis. Inclusion criteria were: i) one isolate per patient; ii) only the referent isolate, in cases of a hospital outbreak; and iii) at least one isolate from every ward participating in the study.

Among the 49 ESBL-producing isolates, 13 were isolated from patients referred to the Pasteur Institute Medical Laboratory and 36 were from hospitalized patients. Distribution of isolates by hospital, ward and specimen is shown in Table 1.

**Table 1:** Distribution of isolates among patient category, ward and specimen types

				**Hospital**				**Ward**						**Specimen**			
**Species**	**No**	**Hospital**	**IPM**	**HJRA**	**HOMI**	**Befelatanana**	**Tsaralalana**	**Surgery**	**Trauma**	**Intensive care**	**Pediatrics**	**Urology**	**Dermato**	**Pus**	**Blood**	**Urine**	Other*
***E. cloacae***	**14**	**12**	**2**	8	2	1	1	2	5	1	3	1	0	9	4	1	0
***E. coli***	**18**	**14**	**4**	12	2	0	0	3	6	3	0	1	1	12	0	4	2
***K. pneumoniae***	**14**	**7**	**7**	4	3	0	0	1	3	3	0	0	0	6	3	5	0
***K. oxytoca***	**3**	**3**	**0**	0	1	1	1	0	0	1	2	0	0	0	3	0	0
***No****(%)*	**49 (%)**	**36** (73.5)	**13** (26.5)	**24** (49)	**8** (16.3)	**2** (4.1)	**2** (4.1)	**6** (12.2)	**14** (28.6)	**8** (16.3)	**5** (10.2)	**2** (4.1)	**1** (2)	**27** (55.1)	**10** (20.4)	**10** (20.4)	**2** (4.1)

*Other: CSF, sputum.

IPM: Pasteur Institute Medical Laboratory.

HJRA: Joseph Ravoahangy Andrianavalona Hospital.

HOMI: Military Hospital.

Antimicrobial susceptibility analyses showed that all isolates were resistant to all the β-lactams used but were susceptible to cefoxitin and imipenem. Resistance to cefoxitin in all *E. cloacae* isolates was due to the inducible production of *AmpC* β-lactamase from a chromosomal gene. All ESBL-producing isolates were also multidrug-resistant and most of them were resistant to: aminoglycosides (87.7% to gentamicin, 93.8% to tobramycin), trimethoprim-sulfamethoxazole (100%) and quinolones (75.5% to nalidixic acid, 69.3% to ciprofloxacin).

### Molecular epidemiology

ERIC-PCR and rep-PCR analyses revealed different restriction patterns for each isolate and showed that they were not clonally related (data not shown).

### Molecular analysis

Nucleotide sequence analysis of the *bla*_CTX*-*M_ and *bla*_SHV_ genes showed that only the CTX-M-15 and SHV-12 genes were present in these isolates. Only TEM-1 and OXA-1 were identified in the TEM- and OXA-producing isolates.

The CTX-M-15 gene was detected in 37 isolates (75.5%) and the SHV-12 gene in 19 (38%). The *ISEcp1* insertion sequence was identified in all 37 *bla*_CTX-M_-carrying isolates. Of the 37 isolates positive for CTX-M-15, ten (27%) also carried only TEM-1, nine (24.3%) also carried only OXA-1, and 16 (43.2%) carried TEM-1 and OXA-1 genes (Table 1). Of the 19 SHV-12-positive isolates, six (31.6%) also carried only TEM-1, four (20.1%) also carried only OXA-1 and six (31.6%) carried TEM-1 and OXA-1 genes (Table 1). Eight isolates (16.3%) (two *E. coli*, five *K. pneumoniae* and one *E. cloacae*) carried both *bla*_CTXM-15_ and *bla*_SHV-12_ and six of these were additionally TEM-1- and OXA-1-positive.

The resistance genes most frequently present were *aac(6*^*′*^*)-Ib* (n=35, 71.4%) (33 were *aac(6*^*′*^*)-Ib-cr,* 67.3%), *sul1 and sul2* (n=25, 51%), *tetA* (n=24, 48.9%), *qnrB* (n=12, 24.5%) and *qnrA* (n=1, 2%). Among the six isolates carrying *bla*_CTXM-15_, *bla*_SHV-12_, *bla*_TEM-1_ and *bla*_OXA-1,_ all of these also carried *aac(6*^*′*^*)-Ib (5 were aac(6*^*′*^*)-Ib-cr)*, *sul1-sul2,* and five harbored *tetA*.

Overall β-lactam resistant isolates harbored β-lactamases genes (CTX-M-15, SHV-12, TEM-1 and/or OXA-1) as well as trimethoprim-sulfamethoxazole resistant isolates sulfamide genes (*sul1* and/or *sul2*). Ten (27.8%) of ciprofloxacin resistant isolates and 3 (25%) of ciprofloxacin susceptible isolates were *qnr* positive. Twenty five (69.2%) of ciprofloxacin resistant isolates and 8 (61.5%) of ciprofloxacin susceptible isolates were *aac(6*^*′*^*)-Ib-cr* positive And, 27 (71%) of amikacin susceptible isolates and 8 (72.7%) of amikacin resistant isolates were *aac(6*^*′*^*)-Ib* positive.

Forty-eight isolates were positive for the class-1 integron gene and it was absent in only one *K. oxytoca* isolate*.* We amplified the class 1 integrons in twenty-five (52%) of these 48 isolates using 5^′^CS and 3^′^CS primers. The sizes of the class 1 integron amplicons, which correspond to the approximate sizes of the cassette regions, were between 0.7 kb and 2 kb. Seven different cassettes were identified, including the *dfr* gene that encodes resistance to trimethoprim and the *aadA* gene that encodes resistance to streptomycin. The two genes most frequently associated with each other were *dfrA17* and *aadA5* (11/25, 22.4%) (Table 2).

**Table 2:** **Characteristics of ESBL-producing *Enterobacteriaceae *isolates and their associated drug resistance genes and gene cassettes**

		**ESBLs**			**Other β-lactamases**		**Associated drug resistance genes**									**Gene cassettes**						
**Species**	**No**	***CTX-M-15***	***SHV-12***	**Both**	***TEM-1***	***OXA-1***	***TetA***	***aac6'-1b***	***aac6'-1b-cr***	***qnrA***	***qnrB***	***catB3***	***sul1***	***sul2***	***sul1- sul2***	***aadA1***	***aadA2***	***aadA4***	***aadA5***	***dfrA5***	***drA22***	***dfrA17*-*aadA5***
*E. coli*	18	14	2	2	12	13	8	14	13	0	3	0	2	3	8	2	1	1	1	2	0	6
*K. pneumoniae*	14	6	3	5	7	13	9	13	13	0	5	4	2	5	7	0	2	0	0	1	1	3
*K. oxytoca*	3	1	2	0	1	0	0	0	0	0	0	0	0	0	2	0	0	0	1	0	0	0
*E. cloacae*	14	8	4	1	12	2	7	8	7	1	4	0	0	6	8	0	1	1	0	0	0	2
Totals	49	29	11	8	32	28	24	35	33	1	12	4	4	14	25	2	4	2	2	3	1	11

### Resistance transfer

Transfer of ESBL by conjugation to *E. coli* J53-2 was successful for 29 (59.2%) of the 49 ESBL isolates, which consisted of eight *E. coli*, eight *E*. *cloacae* and 12 *K. pneumoniae* isolates and one *K. oxytoca* isolate*.* ESBL transfer by plasmid DNA electroporation into *E. coli* DH10B was successful for five (10.2%) of the 20 remaining isolates; four were *E. coli* isolates and one was a *K. pneumoniae* isolate. The presence of *bla*_CTX-M_, *bla*_SHV_, *bla*_TEM_ and *bla*_OXA_ was confirmed by PCR in the 34 transconjugants and transformants. Transfers of non-ESBL resistance genes (tetracycline, gentamicin and trimethoprim-sulfamethoxazole) were also detected by antimicrobial susceptibility testing.

### Plasmid replicon type determination

PCR-based replicon typing in the 34 transconjugants and transformants demonstrated the presence of the IncFII, HI2 and FIA replicons in these isolates (Table 3). IncFII was the most prevalent replicon type and was detected in 20 (58.8%) (10 *E. coli* and 10 *K. pneumoniae*) of the 34 isolates. HI2 was found in 13 (38.2%) isolates (eight *E. cloacae*, three *K. pneumoniae*, one *E. coli* and one *K. oxytoca*) and FIA was found in one *E. coli* isolate. The plasmids carrying *bla*_CTX-M-15_ were assigned to the FII (n=12) and HI2 (n=8) replicon types. Plasmids carrying *bla*_SHV-12_ (n=5) or carrying both *bla*_CTX-M-15_ and *bla*_SHV-12_ (n=2) were assigned to FII.

**Table 3:** β-lactamase genes transferred to transconjugants and electroporants and their replicon type

**β-lactamase genes**	**Replicon type**	**Transconjugants**					**Electroporants**		
		***E. coli***	***K. pneumoniae***	***K. oxytoca***	***E. cloacae***	**Totals**	***E. coli***	***K. pneumoniae***	**Totals**
CTX-M-15	FII	2	1	0	0	3	2	0	2
	HI2	0	1	0	1	2	0	0	
	FIA/FIB	1	0	0	0	1	0	0	
SHV-12	FII	0	3	0	0	3	1	0	1
OXA-1	FII	0	1	0	0	1	0	0	
CTX-M-15+TEM-1	FII	0	0	0	0		1	0	1
	HI2	0	0	1	6	7	0	0	
CTX-M-15+OXA-1	FII	3	3	0	0	6	0	0	
	HI2	1	1	0	0	2	0	0	
SHV-12+TEM-1	FII	1	0	0	0	1	0	0	
TEM-1+OXA-1	HI2	0	0	0	1	1	0	0	
CTX-M-15+SHV-12+TEM-1	FII	0	0	0	0		0	1	1
CTX-M-15+TEM-1+OXA-1	HI2	0	1	0	0	1	0	0	
CTX-M-15+SHV-12+OXA-1	FII	0	1	0	0	1	0	0	
Totals		8	12	1	8	29	4	1	5

**Table 4:** Phylogenetic and virulence factors in the *E. coli* isolates

**Phylogenetic**	**No**	**Specimen**				**Virulence factor**					
**group**		Pus*	**Urine**	**Sputum**	**CSF**	***fyuA***	***iutA***	***sfa***	***IroN***	***Iha***	***traT***
A1	14	11	1	1	1	3	6	0	2	0	14
B1	2	1	1	0	0	1	1	0	0	0	2
B2	1	0	1	0	0	1	1	1	0	1	1
D1	1	0	1	0	0	1	1	0	0	0	1
Totals	18	12	4	1	1	6	9	1	2	1	18

*Deep pus, surgical wounds.

### E. coli phylogenetic groups and virulence factors

Phylogenetic analysis of the 18 *E. coli* isolates revealed four main phylogenetic groups (A1, B1, B2 and D). Most of these isolates belonged to group A1 (77.7%, n=14), 11 of which were isolated from pus. All 18 isolates harbored genes related to complement resistance (*traT*) but none harbored any of the *papG* alleles or the *fimH, afa, hlyA, cnf1, kpsMII* or *sat* genes. Ten isolates from groups A1, B1 and D harbored genes encoding siderophores (*fyuA*, *iutA* and *IroN*) (Table 4).

The single *E. coli* isolate in the B2 group was an O25b-ST131 clone and was isolated from the urine of a hospitalized patient. This *E. coli* isolate harbored *bla*_CTX-M-15_, *tetA*, *aac(6*^*′*^)*-Ib-cr* and *sul1-sul2,* and was assigned to the FII replicon type. Genes encoding siderophore (*fyuA and iutA*) and genes involved in the formation of adhesins (*iha*) or *fimbriae* (*sfa*) were detected in this isolate, but it produced neither cytotoxin nor hemolysin.

## Discussion

We extensively characterized 49 ESBL-producing *Enterobacteriaceae* collected over a period of 15 months in four hospitals and at the Pasteur Institute Medical Laboratory. Previous studies in Antananarivo have shown resistant bacteria clonal diffusion in hospital settings [20,30], but among the 49 non-representative ESBL-producing *Enterobacteriaceae* studied, no clonal isolates have been found.

The *bla*_CTX-M-15_ ESBL gene is considered to be the most prevalent ESBL worldwide [17,18,23,31,32]. We also found *bla*_CTX-M-15_ to be the most prevalent ESBL in Madagascar, as it was detected in 75.5% of the isolates we studied. A study involving nine Asian countries reported that *bla*_CTX-M-15_ was highly prevalent among ESBL-producing *K. pneumoniae* isolates (60%, 55/92) [17]. In Tunisia, Dahmen et al. reported that 91% of the ESBL-producing isolates carried *bla*_CTX-M-15_ genes [23]. Our findings are intermediate between those found in Asia and in Tunisia and confirm the predominance of *bla*_CTX-M-15_ among ESBL-producing isolates. In Antananarivo, a previous study conducted in the neonatal units of two hospitals in 2006 documented that a clonal outbreak of *K. pneumoniae* harbored *bla*_CTX-M-15_ and *bla*_SHV-2_ genes [20]. In 2009, a community-based study of the intestinal carriage of 49 ESBL-producing *Enterobacteriaceae* demonstrated that the most prevalent ESBL gene was *bla*_CTX-M-15_ (93.9%), followed by *bla*_CTX-M-3_, *bla*_SHV-12_ and *bla*_SHV-2a_[33]. The presences of *bla*_CTX-M-15_, *bla*_CTX-M-3_, *bla*_SHV-2_ and *bla*_SHV-12_ is not surprising as molecular analysis indicated that *bla*_CTX-M-15_ derived from *bla*_CTX-M-3_[6] and *bla*_SHV-12_ from *bla*_SHV-2_[34].

CTX-M genes may disseminate through clonal expansion or horizontal gene transfer [35,36]. In our study, *ISEcp1* was found upstream from *bla*_CTX-M-15_ at variable distances, as was previously described [18]. *ISEcp1* was found to be in the vicinity of many *bla*_CTX-M_ genes (including *bla*_CTX-M-15_) and was reported to contain sequences resembling a typical promoter region [11]. Then, plasmids carrying *bla*_CTX-M-15_ were assigned to the IncFII, IncFIA or IncHI2 incompatibility group replicons. Association of the *bla*_CTX-M-15_ gene with IncF plasmids carrying the FII replicon in association with the FIA or FIB replicon has been reported previously for isolates in Canada, France, Spain, Tunisia, and the United Kingdom [35,36]. The first evidence of the association of the FII plasmid with the *bla*_CTX-M-15_ gene was demonstrated by sequencing the entire pC15-1a plasmid from epidemic *E. coli* isolated in Canada [2]. The IncHI2 plasmid, frequently associated with *bla*_CTX-M-2_ or *bla*_CTX-M-9_, was first identified in *Serratia marcescens*[10], but rarely reported in association with *bla*_CTX-M-15_.

Like *bla*_CTX-M-15_, *bla*_SHV-12_ is also widely distributed. In our study, 38% of the isolates harbored *bla*_SHV-12_. First described in Switzerland [37] and subsequently found in various continents, including Africa [38], *bla*_SHV-12_ is most often found in Asia [34]. Plasmids carrying *bla*_SHV-12_ were assigned to the IncFII replicon, as previously reported in France [39]. Evolutionary analysis of GenBank sequences indicated that *bla*_SHV-12_ evolved from the branch of *bla*_SHV-2a_[34]. Although it is possible that this transformation occurred in Antananarivo, as *bla*_SHV-2a_ was reported in neonatal units in 2009 [20]. It can also be assumed that the local emergence of *bla*_SHV-12_ could be explained by introduction of international clones.

Our antimicrobial susceptibility analysis of the ESBL-producing isolates found highly prevalent resistances to gentamicin (87.7%); tobramycin (93.8%); ciprofloxacin (69.3%) and to trimethoprim-sulfamethoxazole (100%) and confirm the presence of multidrug-resistant isolates in Antananarivo [19,22]. The finding of multidrug resistance among ESBL-producing isolates is of great clinical relevance due to the severely limited therapeutic options and the high risk of treatment failure in patients infected with these strains.

Genes encoding ESBLs are often associated with determinants of resistance to other antimicrobial agents, including aminoglycosides (*aac(6)-Ib*), fluoroquinolones (*qnr*), tetracycline (*tetA*), and trimethoprim-sulfamethoxazole (*sul*) and are frequently located on plasmids belonging to the IncF group [10]. In this study, we found the first example in Madagascar of the plasmid-mediated quinolone resistance (PMQR) genes: *qnrB* (24.5%) and *qnrA* (2%), and a variant gene *aac(6*^*′*^*)-Ib-cr* (67.3%) that encodes an aminoglycoside-modifying enzyme. *Qnr* gene prevalence was higher in the *K. pneumoniae* (41.7%) isolates than in the *E. coli* (25%) isolates, which has been noted by other authors [24,40]. The *aac(6*^*′*^*)-Ib-cr* gene accounted for 94.3% (33/35) of the *aac(6*^*′*^*)-Ib* genes detected. This high proportion of *aac(6*^*′*^*)-Ib-cr/aac(6*^*′*^*)-Ib* was also observed in a previous study [40]. The PMQR genes *qnr* and *aac(6*^*′*^*)-Ib-cr* are now recognized to be geographically widespread [24,25]. These genes have been previously reported to be associated with ESBLs. The horizontal transfer of plasmids harboring genes encoding for ESBLs and PMQR genes could have promoted this co-resistance.

The cassette region could not be amplified by PCR in 23 class 1 integron-containing isolates, which may have been due to the lack of the 3^′^CS. The analysis of 25 cassette regions revealed a predominance of *aadA* and *dfrA* genes, which confer resistance to aminoglycosides and trimethoprim, respectively. This result correlates with previous studies of African *Enterobacteriaceae* isolates [27,41]. The combination of *dfrA17-aadA5* (22%) was the one most frequently detected in our study. Similar findings were reported for isolates from Taiwan and Tunisia, as *dfrA17-aadA5* was found in 81 of 224 (36%) and in 3 of 4 (75%) *E. coli* class 1 integrons*,* respectively [42,43].

Analysis of the phylogenetic groups and virulence factors of *E. coli* isolates revealed that most of these isolates belong to group A1. The phylogenetic group A1 consists of commensal enteric *E. coli* and may therefore be the natural reservoir of pathogenic isolates. Pathogenic *E. coli* isolates may have derived from commensal isolates by acquiring chromosomal or extra chromosomal virulence operons [44]. Although virulence determinants are considered to be mobile, strain phylogeny and virulence may be linked [45]. The B2 phylogenetic group, which diverges from the commensal isolates, evolved toward extra intestinal virulence by acquiring numerous pathogenic determinants [12].

We also encountered an *E. coli* isolate belonging to group B2, harboring *bla*_CTX-M-15_ and other resistance genes, and corresponding to the worldwide pandemic clone O25b-ST131. It has been reported that most O25-ST131 isolates are multidrug-resistant, produce CTX-M-15 ESBL enzymes [14] and harbor virulence genes required for pathogenic invasion of hosts. In one study, the genes for adhesins (*iha, fimH*), siderophores (*fyuA, iutA*) and the toxin (*sat*) were found in 95% - 100% of the O25b-ST131 *E. coli* isolates [14], but typical fimbriae and pilus genes, such as those encoded by the *papA* allele, were not. In Africa, few data exist on the presence of ST131. In a South African study, 43% of 23 isolates were ST131 [46]; as were 50% of the CTX-M-15-producing *E. coli* isolates collected in the Central African Republic [13]. The presence of this clone in Antananarivo hospitals is of concern and further studies should be conducted to assess its prevalence.

## Conclusion

Our results highlight the dissemination of multidrug resistant *Enterobacteriaceae* isolates in Antananarivo, in different hospital settings and probably in the community. These findings underline the need for a rational use of antibiotic and for appropriate methods of screening ESBL in routine laboratories in Antananarivo.

## Competing interest

The authors declare that they have no competing interests.

## Authors’ contributions

Conception and design of the study and acquisition of data: HCR, FR, VR, AT, GA. Molecular and genetic studies, molecular analysis: HCR, GA. Analysis of results: HCR, FR, VR, AT, GA. Draft of the manuscript: HCR, FR, BG, AT, GA. Revisiting of the manuscript for important intellectual content: VR, BG, AT and GA. All authors have read and approved the final manuscript.
